# Use of artificial intelligence in critical care: opportunities and obstacles

**DOI:** 10.1186/s13054-024-04860-z

**Published:** 2024-04-08

**Authors:** Michael R. Pinsky, Armando Bedoya, Azra Bihorac, Leo Celi, Matthew Churpek, Nicoleta J. Economou-Zavlanos, Paul Elbers, Suchi Saria, Vincent Liu, Patrick G. Lyons, Benjamin Shickel, Patrick Toral, David Tscholl, Gilles Clermont

**Affiliations:** 1 Department of Critical Care Medicine, School of Medicine, University of Pittsburgh, 638 Scaife Hall, 3550 Terrace Street, Pittsburgh, PA 15261 USA; 2 Department of Medicine, University of Florida College of Medicine Gainesville, Malachowsky Hall, 1889 Museum Road, Suite 2410, Gainesville, FL 32611 USA; 3 Laboratory for Computational Physiology, Massachusetts Institute of Technology, Cambridge, MA 02139 USA; 4 Department of Medicine, University of Wisconsin, 600 Highland Ave, Madison, WI 53792 USA; 5 Algorithm-Based Clinical Decision Support (ABCDS) Oversight, Office of Vice Dean of Data Science, School of Medicine, Duke University, Durham, NC 27705 USA; 6 Department of Intensive Care, Amsterdam UMC, Amsterdam, NL USA; 7 Department of Computer Science, Whiting School of Engineering, Johns Hopkins Medical Institutions, Johns Hopkins University, 333 Malone Hall, 300 Wolfe Street, Baltimore, MD USA; 8 Department of Medicine, Oregon Health & Science University, 3181 S.W. Sam Jackson Park Road, Mail Code UHN67, Portland, OR 97239-3098 USA; 9 Institute of Anesthesiology, University Hospital Zurich, University of Zurich, Frauenklinikstrasse 10, 8091 Zurich, Switzerland; 10 Division of Pulmonary Critical Care Medicine, Duke University School of Medicine, Durham, NC 27713 USA; 11 Amsterdam UMC, ZH.7D.167, De Boelelaan 1117, 1081 HV Amsterdam, The Netherlands; 12 2000 Broadway, Oakland, CA 94612 USA; 13 Amsterdam UMC, ZH.7D.165, De Boelelaan 1117, 1081 HV Amsterdam, The Netherlands; 14 VA Pittsburgh Health System, 131A Building 30, 4100 Allequippa St, Pittsburgh, PA 15240 USA; 15 Department of Medicine, Johns Hopkins School of Medicine, AI and Health Lab, Johns Hopkins University, Baltimore, MD USA; 16 Bayesian Health, New york, NY 10282 USA

**Keywords:** Complexity, Healthcare policy, Machine learning, Predictive analytics, Systems engineering

## Abstract

**Background:**

Perhaps nowhere else in the healthcare system than in the intensive care unit environment are the challenges to create useful models with direct time-critical clinical applications more relevant and the obstacles to achieving those goals more massive. Machine learning-based artificial intelligence (AI) techniques to define states and predict future events are commonplace activities of modern life. However, their penetration into acute care medicine has been slow, stuttering and uneven. Major obstacles to widespread effective application of AI approaches to the real-time care of the critically ill patient exist and need to be addressed.

**Main body:**

Clinical decision support systems (CDSSs) in acute and critical care environments support clinicians, not replace them at the bedside. As will be discussed in this review, the reasons are many and include the immaturity of AI-based systems to have situational awareness, the fundamental bias in many large databases that do not reflect the target population of patient being treated making fairness an important issue to address and technical barriers to the timely access to valid data and its display in a fashion useful for clinical workflow. The inherent “black-box” nature of many predictive algorithms and CDSS makes trustworthiness and acceptance by the medical community difficult. Logistically, collating and curating in real-time multidimensional data streams of various sources needed to inform the algorithms and ultimately display relevant clinical decisions support format that adapt to individual patient responses and signatures represent the efferent limb of these systems and is often ignored during initial validation efforts. Similarly, legal and commercial barriers to the access to many existing clinical databases limit studies to address fairness and generalizability of predictive models and management tools.

**Conclusions:**

AI-based CDSS are evolving and are here to stay. It is our obligation to be good shepherds of their use and further development.

## Introduction

With the advent of increasingly available high-dimensional health data combined with accelerating computational abilities to process and analyze them, there is an emerging opportunity to define health and disease states and their underlying physiologic and pathophysiologic mechanisms with more clarity, precision, and efficiency. Aspirationally, these advances might be applied to real-time diagnosis and patient management. Perhaps nowhere else in the healthcare system than in the intensive care unit (ICU) environment are the challenges to create useful models with direct time-critical clinical applications more relevant and the obstacles to achieving those goals more massive. Machine learning (ML)-based artificial intelligence (AI) techniques to define states and predict future events are commonplace activities in almost all aspects of modern life. However, their penetration into acute care medicine has been slow, stuttering and uneven. There are many papers describing the various types of ML approaches available [1–3]. But the realization of such approaches and tools to aid clinicians has been erratic.

Major obstacles to widespread effective application of AI approaches to real-time care of critically ill patients need to be addressed. Presently, clinical decision support systems (CDSS) cannot replace bedside clinicians in acute and critical care environments. The reasons are many and include the immaturity of CDSS to have situational awareness, the fundamental bias in many large databases that do not reflect target populations of patient being treated (making fairness an important issue), and technical barriers to timely access to valid data and its display in a fashion useful for clinical workflow. The inherent “black-box” nature of many predictive algorithms and CDSS makes trustworthiness and acceptance by the medical community difficult. Logistically, collating and curating in real-time multidimensional data streams of various sources needed to inform the algorithms and ultimately display relevant clinical decisions support format that adapt to individual patient responses and signatures represent the efferent limb of these systems and is often ignored during initial validation efforts. Similarly, legal and commercial barriers to the access to many existing clinical databases limit studies to address fairness and generalizability of predictive models and management tools. We will explore the barriers to effective use of AI in critical care medicine, and ways that either bypass them or address them to achieve effective CDSS.

### Real-world clinical data for both model-building and CDSS

Large amounts of highly granular data—such as those from devices for monitoring and life support, laboratory and imaging studies, and clinical notes—are continuously being generated and stored in electronic health records (EHRs) from critically ill patients. The massive number of patients with available data for analysis dwarfs clinical trial sample sizes. Thus, there is both ample availability of data and a clear opportunity for data-driven CDSS. Compared with clinical trials or prospectively, enrolled cohort studies, disadvantages of real-world data such as bias and non-random missingness, if addressed, are offset by obvious advantages including an unselected patient population with larger sample size and the ability to update and focus analyses, all with the potential to maximize external validity for a fraction of the cost. Currently, most critical care EHR data are only available for patient care and not for secondary use. Barriers include legal and ethical issues related to privacy protection as well as technical issues related to concept mapping across different-based Intensive Care Unit Data (EHR vendors where similar clinical concepts are represented differently, thus introducing semantic ambiguity [4]). But a very large obstacle is the lack of incentive to make intensive care data available for local, regional, or general use. However, the concept that the healthcare system could learn from all data of all their patients is attractive and should foster data solidarity.

Responsible sharing of large ICU datasets at all levels implies finding the right balance between privacy protections and data usability. This requires careful combinations of governance policies and technical measures for de-identification to comply with ethical and legal standards and privacy laws and regulations (e.g., Health Insurance Portability and Accountability Act in the USA and General Data Protection Regulation in the EU). These challenges contributed to the fact that until recently, freely available ICU databases were sourced only from the USA. A partial list of publicly available US, Europe and China large intensive care databases is provided in Table 1. Most are described and accessible on the Physionet platform [5]. There are also numerous databases and data sharing initiatives that are less freely available. Access to these typically requires collaboration with institutes from which the data has been sourced (e.g., Critical Care Health Informatic Consortium, Dutch Data Warehouse and ICUdata.nl).

**Table 1:** Publicly available ICU databases

Databases	Features	Link
MIMIC III	EHR, notes, high-frequency physiology; ICU	https://physionet.org/content/mimiciii/1.4/
MIMIC IV	EHR, notes, high-frequency physiology, electrocardiograms, radiologic images, EEG, Echocardiograms; Emergency department, Hospital, ICU	https://physionet.org/content/mimiciv/2.2/
eICU	EHR; ICU	https://physionet.org/content/eicu-crd/2.0/
AmsterdamUMCdb	EHR; ICU	https://amsterdammedicaldatascience.nl/amsterdamumcdb/
HiRID	EHR, high-frequency physiology; ICU; COVI 19 focused	https://physionet.org/content/hirid/1.1.1/
SICdb	EHR; high-frequency physiology; ICU	https://physionet.org/content/sicdb/1.0.6/
Zhejiang	EHR, ICU	https://physionet.org/content/zhejiang-ehr-critical-care/1.0/
Pediatric Intensive Care	EHR, ICU	https://physionet.org/content/picdb/1.1.0/

EHR, the entire electronic health records of a subject; ICU, the specific EHR of subjects within an ICU exposure; MIMIC, medical information mart for intensive care; ICU, intensive care unit

Operationally, the research question should determine the choice of dataset, as they differ substantially in cohort size, data granularity, treatment intensity, and outcomes. To foster model generalizability, at least two different datasets should be used. One barrier to this kind of external validation would be removed if these free databases were available in common data models using standard vocabularies; the recent effort to map the MIMIC-IV dataset to the Observational Medical Outcomes Partnership (OMOP) common data model is an important first step in this effort [6]. The US-based Patient-Focused Collaborative Hospital Repository Uniting Standards (CHoRUS) for Equitable AI has initiated the generation of a harmonized, geographically diverse, large multi-domain dataset of ICU patients including EHR, text, images and waveform data (bridge2ai.org/chorus). This public-facing dataset should soon be available to complement existing databases with the added advantage of significant diversity. Alternatively, the R-based Intensive Care Unit Data (RICU) and the Yet another ICU benchmark (YAIB) offer opportunities for combined analyses of critical care datasets. Another limitation of these datasets may be their limit of ICU-only data.

Despite the limited number of ICU datasets, the flurry of excellent modeling work afforded by these freely available intensive care datasets has exposed a severe translational gap, with implementation at the bedside and demonstration of improved patient outcomes using those models proving very challenging [7, 8].

### Bias in database origins and model validation/governance

There is a fundamental flaw in building AI-CDSS using existing EHRs and evaluating the models using accuracy against real-world data given existing health disparities present in these databases. This is setup for encoding structural inequities in the algorithms, thereby legitimizing their existence and perpetuating them in a data-driven healthcare delivery system. Social patterning of the data generation process [9] and social determinants of care [10]. The social patterning of data generation pertains to how a patient is represented as her data during healthcare encounters. In an ideal world, everyone is cared for “equitable fashion”. But existing EHRs suffer from bias because of how patients and their care are captured. These biases are reflected and may be reinforced by AI as the processes of model development and deployment. Furthermore, models built on EHR data skewed toward a primarily Caucasian class of patients may not model well African-American, Hispanic or oriental patients [11, 12]. EHR databases that are representative on the demographics of the patients for whom the AI-CDSS is being directed are necessary.

To avert AI-legitimized and AI-enabled further marginalization of those already disproportionately burdened by disease and societal inequities, regulatory guardrails are needed. Such guardrails would be policies and/or incentive structures developed through continuous open dialogue and engagement with communities that are disproportionately burdened, marginalized, or non-represented. But unless the ML community prioritize the “who”—who are developing and deploying AI—and the “how”—is there transparency and accountability for responsible AI, then these CDSS efforts will be less effective.

### Designing clinical decision support systems for situational awareness

Situational awareness (SA) is foundational to decisions and actions in sectors like aviation and medicine [13]. Robust SA is a prerequisite for sound decisions that recognize the relevant elements in an environment, understand their meaning, and forecast their short-term progression. Lapses in SA are a primary cause of safety-related incidents and accidents [13, 14]. SA continuously evolves, influenced by changing external circumstances and individual internal factors. Heavy workloads and fatigue with diminishing mental capacity can hinder a clinician’s ability to achieve and maintain SA in critical care environments. In contrast, having extensive experience in a specific context can enhance SA, as familiarity guides what to focus on. Well-designed CDSS should improve SA.

Presently, AI-based CDSS will work alongside human decision makers as opposed to as autonomous support systems. Such CDSS should transfer essential information to decision makers as quickly as possible and with the lowest possible cognitive effort [15]. User-centered, SA-oriented design is needed for the successful implementation of AI-CDSS. In complex and dynamic environments, AI-CDSS design should allow staff to clearly grasp information, reduce their workload, and strengthen their confidence in the diagnoses, importantly because these aspects promote staff acceptance and trust ultimately determining whether AI-CDSS are implemented.

A wide gap exists between health AI done right and implementations in practice. Building and deploying AI predictive tools in health care is not easy. The data are messy and challenging, and creating models that can integrate, adapt, and analyze this type of data requires a deep understanding of the latest ML strategies and employ these strategies effectively. Presently, only few AI-based algorithms have shown evidence for improved clinician performance or patient outcomes in clinical studies [6, 16, 17]. Reasons proposed for this so-called AI chasm [18] are lack of necessary expertise needed for translating a tool into practice, lack of funding available for translation, underappreciation of clinical research as a translation mechanism, disregard for the potential value of the early stages of clinical evaluation and the analysis of human factors [19], and poor reporting and evaluations [2, 8, 20].

State-of-the-art tools and best practices exist for performing rigorous evaluations. For instance, the Developmental and Exploratory Clinical Investigations of DEcision support systems driven by Artificial Intelligence (DECIDE-AI) [15] guideline provides an actionable checklist of minimal reporting items facilitating the appraisal of CDS studies and their findings replicability. Early-stage clinical evaluation of AI-CDSS should also place a strong emphasis on validation of performance and safety, similar to pharmaceutical trials phase 1 and 2, before efficacy evaluation at scale in phase 3. Small changes in the distribution of the underlying data between the algorithm training and clinical evaluation populations (i.e., dataset shift) can lead to substantial variation in clinical performance and expose patients to potential unexpected harm [21, 22]. Human factor (or ergonomics) evaluations are commonly conducted in safety–critical fields such as aviation, military, and energy sectors [23] evaluating the effect of a device or procedure on their users’ physical and cognitive performance [24]. However, few clinical AI studies have reported on the evaluation of human factors [25]. The FDA recently released the “Artificial Intelligence and Machine Learning (AI/ML) Software as a Medical Device Action Plan,” which outlines their direction [24, 26] and the National Academy of Medicine has announced the AI Code of Conduct [27] but more work needs to be done. Clinical AI algorithms should be given the same rigorous scrutiny as drugs and medical devices undergoing clinical trials.

### Bridging the implementation gap in acute care environments

Timely interventions require early and accurate identification of patients who may benefit from them. Two prominent examples relevant to critical care are models using readily available EHR data that can accurately predict clinical deterioration and sepsis hours before they occur [28–33]; these models exemplify real-time CDSS that alert clinicians and prompt evaluation, testing, and interventions [33]. Translation of these approaches in clinical intervention studies has improved outcomes [16, 34, 35]. Despite these systems’ early promise, important technical and social obstacles must be addressed to ensure their success. Indeed, the previously described “implementation gap” for medical AI extends to predicting clinical deterioration and sepsis CDSS [7].

Most CDSS development begins with retrospective data; these data often have different quality and availability than data in the production EHR, which can degrade model performance during implementation [36, 37]. Further, outcome labels based on EHR data are generally proxies for real-world outcomes. Imprecise retrospective definitions unavailable in real time, such as billing codes, may complicate the validity of outcome labels [38, 39].

The clinical deterioration and sepsis CDSS models generated headlines for their high discrimination. While discrimination is important, more nuance is needed to understand whether a model is “good enough” to be used for individual patient decision-making. Even when discrimination is high, the threshold chosen for alerts may result in suboptimal sensitivity or excessive false alarms [40]. Balancing sensitivity with false alarms and lead time for alerts remains a persistent challenge, and the optimal balance varies by use case [41]. Also, performance variation across settings, case mix and time must be measured and addressed [42, 43]. Evaluating model fairness across socioeconomic groups is another critical consideration before model implementation.

Information Technology infrastructure and expertise are also essential for implementing CDSS effectively. Vendors increasingly provide proprietary “turnkey” CDSS solutions for identifying clinical deterioration and sepsis [42–44]. While convenient, limitations include inconsistent transparency and performance, user experience constraints, and opportunity costs [45]. Alternative approaches may improve performance but generally require substantial resources and may be more vulnerable to “break-fix” issues and other challenges [46].

The social challenges to CDSS implementation are substantial. Successful implementation requires an understanding of intended users, their workflows and resources, and a vision of how these should change based on CDSS output [47, 48]. Implementation science methods offer guidance. Formative work might use determinant frameworks and logic models to understand which behaviors a CDSS is meant to influence, thereby informing clinical workflow [49, 50].

Efforts to comprehend expected user needs may raise trust and facilitate adoption. Model explainability also improves trust and CDSS adoption. The high complexity of many “black-box” ML models may preclude clinicians from valuing CDSS information when the output is incongruent with clinical intuition. Modern approaches to improving explainability include SHapley Additive exPlanations, a model-agnostic approach to visualizing predictor variable contributions to model output based on game theory. User interface design for real-time CDSS requires expertise in human factors, could be limited by vendor software capabilities and may require adherence to regulatory guidance by governmental agencies.

CDSS must be paired with the ability to measure what matters to patients and clinicians. Evaluation frameworks from implementation science may facilitate CDSS evaluations, capturing elements of both efficacy and effectiveness [51]. Study design choices for implementation evaluation will depend on available resources, local factors, and the clinical problem. Pragmatic randomized trials and quasi-experimental designs offer advantages over pre-post designs or comparisons against historical controls [34, 52].

### A roadmap to effective adoption of AI-based tools

**Fig. 1:**
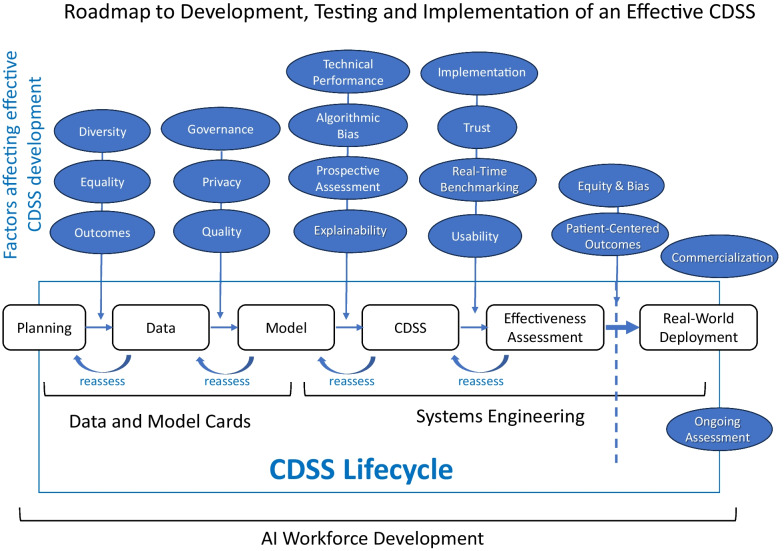
The process of creating, testings, and launching an effective Clinical Decision Support System (CDSS) is multifaceted and ongoing. The interaction of multiple processes and involvement of various stakeholders along the way improve the likelihood of final adoption during real-world deployment (dashed vertical line). Importantly, as illustrated in this work flow diagram, is ongoing assessment refining models and information transfer options. At the start one uses a model card which is a short document that provides key information about a machine learning model. This is central to maintaining focus throughout the workflow cycle of CDSS development

The integration of AI into healthcare necessitates meticulous planning, active stakeholder involvement, rigorous validation, and continuous monitoring, including the monitoring of adoption. Adhering to software development principles and involving end-users enables CDSS to ensure successful adoption, ultimately resulting in improved patient care and enhanced operational efficiency. A dynamic approach that involves regular assessment and refinement of AI technology is essential to align it with evolving healthcare needs and technological advancements. Creating data cards, which are structured summaries of the essential facts about various aspects of the ML datasets needed by stakeholders across a project’s lifecycle for responsible CDSS development, is a very useful in insightful initial step in this process. Figure 1 summarizes the issues address in this paper as a roadmap to effective CDSS completion, and Table 2 itemizes obstacles to efficient CDSS roll out and potential solutions.

**Table 2:** Guide to Addressing Obstacles in CDSS Development

Phase of development	Obstacle	Cause	Work around	Aspirational solution
Planning	Identify ideal source population	Too narrow a target population Inappropriate selection of outcome measures	Involve a diverse group of stakeholders with different perspectives to identify shortcomings and goals	Involve diverse stakeholders to plan data collection
	Inherent bias in data	Limited number of variables included in the data source Limited availability of relevant data elements (e.g., Social Determinants of Health) Poor quality of data dictionary	Produce data cards Identify non-traditional sources of data Improve data dictionaries	Ongoing construction of large, diverse, bias-aware, multimodal datasets
	Data source has limited diversity and marginally relevant to target population	Limited number of large databases available Poor choice of source data	Identify additional data sources Propensity matching	
	Variability in source data content and formatting	Homegrown, narrowly focused data dictionaries and case report forms No concern for reuse	Post-hoc harmonization of data	Adoption of a standard common data model for harmonization
Data	Data source identification	Existing data not appropriate for research question Data is difficult to find	Use and modify data that can be found	Large data distribution platforms
	Data access	Unclear governance Data may be impossible to access	Data use agreements Document investigators’ qualifications, giving tiered access to data	Transparent and uniform governance
	Privacy	Heterogeneity of privacy rules Technical issues with data sharing Lack of guidelines	Awareness of regional privacy requirements Adoption of sufficient technical solution to fulfill these requirements Contract technical expertise	Democratized low-cost technical solutions
	Data quality	Poor planning Poor collection methodology	Data imputation Limit inference	Adoption of existing tools Adoption of guidelines on data quality assessment Develop guidelines for emerging domains of data
	Data Reuse	Limited use of common data models	Post-hoc harmonization Promote easy access	Abide by FAIR principles (www.go-fair.org)
Model development	Technical performance	Choice of performance metric inappropriate for intended CDSS use Risk of over-fitting and lack of generalizability	Model cards Follow existing guidelines for reporting AI model performance	Good AI methodology stewardship
	Algorithmic bias	Inherent or pre-processing bias in training dataset Possible bias not assessed Tools for bias assessment may be incomplete	Use available tools to assess bias at different stages Tune models on subgroups on interest	Guidelines on multi-stage bias assessment Disseminate known limits, shortcomings of CDSS
	Trust and Explainability	Lack of trust in black box models Poor performance of explainable models Output not seen as useful or valuable by end-users	Expanding sets of methods to explain models Chose explainable models whenever decrease in performance not clinically relevant Engage end-users very early in the CDSS development	Team development from initiation to completion of model development
	Prospective evaluation	Technical access to relevant real-time data Governance barriers to real-time data Latency in accessing real-time data	Simulate real-time data arrival Silent deployment	Real-time EHR integration for deployment in a learning healthcare system
CDSS	Real-time benchmarking	Technical obstacles to deliver model prediction to bedside Governance-based obstacles to bedside deployment Ethical issues with real-time deployment	Early involvement of key administrative, technical, and clinical stakeholders	Flexible pipeline for scalable expansion of tools Improved technical standards for real-time integration of AI applications with EHR vendors Wide adoption of such standards
	Transitioning a model into a CDSS	Human factor engineering Implementation	Involvement of end-users in system design Choosing appropriate metrics of performance System usability and technology acceptance evaluations	Systematic usability and acceptability assessments Periodic reassessments
Effectiveness assessment	Measuring impact	Impact on clinical outcomes Impact on ICU processes Impact on technical team	Choosing appropriate clinical outcomes Measure resource use Choosing appropriate technical outcomes	Engage stakeholder to define a process for technology effectiveness assessments National guidance for technology effectiveness assessment Ongoing compliance assessment
	Adoption	Compliance	Identify internal “super users” to lead adoption Involve end-users in evaluation	
	Unclear return on investment	Impact of economic outcomes	Engage financial decision makers early in system design Collect data that allows economic assessment	
Real-world deployment	CDSS degrading performance over time	Patient-specific issues changing or care giver support decreasing	Review model performance and address changes in input variables	Continual CDSS model performance evaluation
			Modify CDSS to be more ergonomic for caregivers Re-educate caregivers	
	Commercial availability	Many solutions are homegrown Implementation gap and lack of practical solutions FDA, GDPR clearance	Clinicians’ involvement in product development and testing AI-technology assessment bodies in Health system that prioritize development	Evolving national guidance on product development and commercial clearance Inherent cyber security of EHR-linked algorithms used in modeling
All phases	Limited technical expertise	Inherent lack of expertise of clinicians in big data manipulation and model development Siloed development	Build interdisciplinary teams Broadly available educational opportunities	Basic training in AI in clinical curricula Early engagement of potential stakeholders Worldwide datathons
	Limited scalability	Solution not applicable outside ICUs or in resource limited environments	Conceive of critical illness as a continuum and formulate outcomes accordingly Prioritize low-technology solutions	

It is vital that to have a designated owner to define the problem the AI technology is intended to solve and oversee the design and deployment [53]. Involving a broader array of stakeholders before the pilot phase is equally crucial. This inclusive approach encourages early feedback and insights before full deployment, enhancing potential adoption and ensuring effective communication with the owner throughout the pilot deployment. Engaging a representative team of stakeholders deepens their understanding of the technology and its seamless integration into existing workflows. This involvement should encompass a diverse range of end-users, including medical professionals, clinicians, patients, caregivers and other stakeholders within the clinical workflow. Early and active engagement in design processes by stakeholders ensures alignment of technology with its intended objectives, its smooth integration into established healthcare processes, and early prevention of safety risks and biases.

User Acceptance Testing forms a critical step in the software qualification process, where end-users rigorously evaluate the technology's functionality and, in the case of CDSS, agreement with the model output. It can inform false-positive and false-negative risks. This evaluation ensures that technology aligns with their specific needs and expectations. The User Acceptance Testing phase offers invaluable insights into requirements, integration options and validation of AI-CDSS outputs based on those requirements and contributes significantly to interface design improvements. Human factor studies can be performed to demonstrate technology usability [54]. Usability factors and empirical measures can also be used in the testing phase [55]. By involving end-users in testing, the technology meeting its intended use is greatly facilitated and a sense of ownership is cultivated, empowering end-users with a deeper understanding of how the technology integrates into their workflow, further enhancing its overall effectiveness. Before the AI-CDSS is introduced into the workflow, needs-adjusted training can facilitate AI-CDSS acceptance and instructions for its use [56].

Effectively measuring and monitoring AI technology adoption is pivotal for evaluating its real-world effectiveness and pinpointing areas for enhancement. Utilizing quantitative metrics, such as tracking interface interactions like button clicks, provides data on user engagement, shedding light on usage patterns. Concurrently, surveys and qualitative interviews, focus groups, and direct observation offer deeper insights into user experiences and perceptions. This dual approach enables healthcare organizations to refine the technology, prioritizing user satisfaction and feedback [57]. It also serves as an avenue for end-users to voice safety concerns and broader issues. Real-world deployment necessitates a consistent feedback mechanism, since end-users might override recommended actions or decisions or disagree with AI-CDSS output. This feedback should be systematically shared with the development team or relevant organization, capturing information on agreement with the technology’s output and recommended decisions or actions. This process is akin to documenting protocol deviations in clinical trials and should encompass any safety concerns or other issues, such as bias. A comprehensive root cause analysis of disagreements, along with mitigation strategies, should be recorded at the point of care, enhancing the overall safety and efficacy of the technology.

The transition from the pilot phase to general deployment marks a pivotal stage in AI adoption. Successful pilot deployments act as a springboard for broader adoption [58]. Human trust is an important factor, and further education on AI and transparency information can build this trust for clinicians and patients [59–61]. Identifying and leveraging technology champions within the healthcare system can profoundly influence the dissemination of the technology’s value. These advocates play a vital role in communication campaigns, training, and facilitating a seamless transition to widespread deployment, ensuring a comprehensive understanding of the technology’s benefits.

### Governance and regulatory considerations

The rapid advances in AI, and in particular the release of publicly available generative AI applications leveraging advanced large language models, have greatly accelerated discussions considering the promises and pitfalls related to AI deployment in society and healthcare [7–17]. Heightened concerns about the development and deployment of AI have generated discussion about how to ensure that AI remains ‘aligned’ with human objectives and interests. As a result, a rapidly evolving set of regulations are being drafted by a wide variety of regional, federal, and international governing bodies that are expected to become formalized over the next three years, such as the World Health Organization’s report on the Ethics and Governance of AI for Health and the European Union’s report on AI in Healthcare: Applications, Risks, and Ethical and Societal Impacts. In the USA, the White House’s Blueprint for an AI Bill of Rights: Making Automated Systems Work for the American People; the National Institute of Standards and Technologies’ Artificial Intelligence Risk Management Framework; and the Food and Drug Administration’s guidance on Software as a Medical Device and Clinical Decision Support Devices do the same. These documents highlight several governing principles for safe AI including that the technology should: do no harm; be safe; be accurate; be free of bias and discrimination; preserve privacy; be intelligible to end-users; be monitored on an ongoing basis; and address consent for use. These principles follow closely with effective, safe, and equitable healthcare delivery, yet AI poses novel challenges given its dependence on rapidly evolving and increasingly complex algorithmic underpinnings.

### The AI workforce

The rapid growth of AI has accelerated discoveries across diverse scientific fields and affected every work environment [62–65] and is reshaping the labor market with unprecedented speed and scale, with 40% of the global workforce expected to require AI, enhancing the need for significant AI upskilling or reskilling [66]. The rapid adoption of AI into healthcare and clinical research is an opportunity to transform how we discover, diagnose, treat, and understand health and disease. The American Medical Association supports this vision of human–machine collaboration by rebranding the AI acronym as “augmented intelligence” [67]. AI-augmented clinical care requires an AI-literate medical workforce, but we presently lack sufficiently skilled workers in medical domain-specific AI applications. Many biomedical and clinical science domain experts lack the foundational understanding of AI systems and methodologies. There is currently not enough opportunity for rapid AI training in clinical medicine and research. AI tools and systems require increasingly less underlying mathematical or technical knowledge to operate, aligning with the US Food and Drug Administration (FDA) processes to authorized AI algorithms as “software as a medical device” [68]. Evidenced by NIH Common Fund Programs (AIM Ahead and Bridge2AI), there is a universally acknowledged AI training gap and a clear need for accessible and scalable AI upskilling approaches to help raise the first global generation of AI-ready healthcare providers.

The future ICU workforce will require specialized AI critical care training that prioritizes a conceptual AI framework and high-level taxonomies over programming and mathematics. Clinicians must understand the indications and contraindications of relevant clinical AI models, including the ability to interpret and appraise published models and training datasheets associated with a given AI tool across various demographic populations [69, 70]. AI training programs in critical care must also be agile enough to adapt to rapid shifts in the AI landscape. Last, these programs should instill in trainees a fundamental working knowledge of bias, fairness, trust, explainability, data provenance, and responsibility and accountability.

It is essential that the diversity of AI researchers mirrors the diverse populations they serve. There are significant gaps in gender, race, and ethnicity [71, 72] Lack of diverse perspectives can negatively impact resulting products, as has plagued the AI field for years [73, 74]. The 2022 Artificial Intelligence Index Report states that 80% of new computer science PhDs specializing in AI were male, and 57% were White, which has not changed significantly since 2010. There is thus a critical need for a nationwide academic-industrial collaborative training programs to fund, develop, and mentor diverse AI researchers to ensure AI fairness in biomedical research [75].

## Conclusion

AI is here to stay. It will permeate the practice of critical care and has immense potential to support clinical decision making, alleviate clinical burden, educate clinicians and patients, and save lives. Yet, although this complex, multifaceted, and rapidly advancing technology will reshape how healthcare is provided, it brings along deep ethical, fairness, and governance issues that must be addressed in a timely fashion.

## Data Availability

NA.
